# Metabolomics and Agriculture: What Can Be Done?

**DOI:** 10.1128/mSystems.00156-17

**Published:** 2018-03-06

**Authors:** Rodolpho Martin do Prado, Carla Porto, Estela Nunes, Claudio Lima de Aguiar, Eduardo Jorge Pilau

**Affiliations:** a Laboratório de Biomoléculas e Espectrometria de Massas—Labiomass, Departamento de Química, Universidade Estadual de Maringá, Maringá, Paraná, Brazil; b Embrapa Soja, Rodovia Carlos João Strass, s/n, Acesso Orlando Amaral, Distrito de Warta, Londrina, Paraná, Brazil; c Laboratório Hugot de Tecnologia em Sucroderivados, Escola Superior de Agricultura Luiz de Queiroz, Universidade de São Paulo, Piracicaba, São Paulo, Brazil

**Keywords:** GNPS, agriculture, livestock, mass spectrometry, metabolomics

## Abstract

The importance of Brazil as a producer and exporter of food and feed will continuously increase. Despite the recent economic and political problems in Brazil, the scientific field is expanding. Cutting-edge technology has only recently become available in the country, and we can now also join efforts with the global community to tackle global challenges. Using metabolomics based on mass spectrometry approaches to understand system-wide metabolism and metabolic pathways can have a significant impact in the society. With the recent development of a platform for organization and sharing of tandem mass spectrometry data, the global community can now work with complex biological samples. In this Perspective, we aim to describe how challenges and problems in global and local agriculture can be addressed using metabolomics based on mass spectrometry strategies.

## Abstract (precis)

The importance of Brazil as a producer and exporter of food and feed will continuously increase. Despite the recent economic and political problems in Brazil, the scientific field is expanding.

## PERSPECTIVE

Current estimates are that by 2050, human population will peak and food consumption will double compared to 2005 to 2007 (1). Increasing the yield of crops is likely to be the driver to address such challenges; however, the average yield increase of crops will be insufficient to feed a population of 9.6 billion people by 2050 (2). Additionally, exploring new lands is another solution to increase food production, but there is a high environmental cost (although it is difficult to measure and to predict the impacts that will occur both locally and globally). Furthermore, managing not only the quantity of food but also food safety will be another challenge in the upcoming years, especially with respect to chemical and microbial contaminants and food traceability (3,4). Finally, agriculture and animal production will continue to be pressured by society, as these activities contributed 24% of global greenhouse gas emissions in 2010 (5).

Brazil is a developing country and yet is the world’s second largest agricultural exporter (6). In part, this is due to its large farmland areas (283 million ha) and to the continuous increase in productivity. Indeed, agricultural output and livestock output have doubled and tripled, respectively, compared to 1990 (6). Furthermore, research on tropical agriculture, including such topics as nitrogen fixation, novel new grain varieties, and livestock breeds, has also been an important aspect of the development of Brazilian agriculture. However, there is still a necessity to understand fundamentals of agriculture, such as the metabolism of plant, insects, and microorganisms, and how they interrelate.

Recent advances of metabolomics based on a mass spectrometry (MS) approach are due to the development of data repositories, where the community can curate large data sets that can be analyzed in a manner faster than manual analysis. Thus, complex samples, enriched or not enriched in chemical entities, can be analyzed. Since analysis using MS coupled to liquid chromatography (LC-MS) has the capacity to detect thousands of ions from a single sample, metabolomics approaches based on LC-MS can generate a chemical inventory with a great diversity of metabolites associated with biological samples, such as microbes, plants, and other organisms (7). The main challenge for such metabolomics-based strategies is to identify both known and unknown molecules in complex samples.

In this context, experiments using the tandem MS (MS/MS) mode (molecular ion fragmentation) can extensively explore metabolomics dereplication, since the fragments generated are definitive characteristics of a molecule. Thus, experiments using the MS/MS mode are more discriminatory than those just using the ion molecular mass. Consequently, the molecular networking methodology can help in the pursuit of analysis of metabolites. The introduction of the online tool Global Natural Products Social Molecular Networking (GNPS), a crowd-sourced knowledge repository and analysis infrastructure, promotes the capture of mass spectrometry expertise from the scientific community. This online platform was recently launched (8) and has started to address such aforementioned issues using analysis based on molecular networking, clustering MS/MS spectra based on spectral similarity. This approach is being widely used in research communities in a collaborative way, mainly by natural product and metabolomics researchers.

Metabolomics in livestock production is an emerging research field, and there is interest in prospecting biomarkers for specific traits such as weight gain, milk quality, methane emission, and health. Studies can use aliquots sampled noninvasively, routinely, and usually with low cost compared to other techniques. The first study to characterize the metabolome of rumen fluid was published only 5 years ago by Saleem et al. (9). Recently, Artegoitia et al. (10) used untargeted metabolomics to analyze the rumen content of steers and observed different metabolites when animals had similar dry matter intakes but different average levels of daily gain. Sun et al. (11) studied the metabolome of rumen fluid, blood, milk, and urine of dairy cows and observed potential biomarkers for milk productivity and quality. Although there have been only a few such studies, the results are promising. In this field, our group is evaluating various extraction methods to characterize the rumen fluid using untargeted metabolomics, in a system-wide metabolism approach. We observed an abundance of metabolites (unpublished data) which was richer than we expected. Metabolomics is also being used to address antimicrobial resistance, a global threat to humans, as the corresponding mortality rates are predicted to be higher than cancer mortality rates by 2050 (12). Agriculture plays a central role in this topic, as it consumes close to two-thirds of antibiotics produced. We expect that a high-throughput pipeline to detect antimicrobial-resistant bacteria using metabolomics will be available in the coming years. For example, bacterial secondary metabolite profiles of antibiotic-resistant mutants were different from those of their progenitors (13). We have an ongoing field study that is being performed with external and internal collaborators to provide a snapshot of antimicrobial resistance in Brazilian farms, using sequencing and the metabolomic approach. As mentioned before, we believe that Brazil will play a central role for livestock production; thus, surveillance of antimicrobial resistance is of global interest.

Asian soybean rust is mainly caused by *Phakopsora pachyrhizi*, which rapidly destroys the soybean plant (14). The disease can cause losses of 10% to 75% of the soybean crop area. Understanding the factors that regulate soybean infection and resistance is an important approach to mitigate losses, and yet there are no studies examining the molecular responses of soybean to *P. pachyrhizi* infection. Furthermore, metabolic information can be used to assist breeding efforts aimed at the development of cultivars with improved resistance to Asian soybean rust. We are addressing the Asian soybean rust in collaboration with the Brazilian Agricultural Research Corporation (Embrapa Soybean, Londrina-PR, Brazil). We are using metabolomics to elucidate the metabolome of soybean plants with respect to stressors, and we are also evaluating fungi involved in the pathogenesis of Asian soybean rust. The GNPS approach will be used to investigate whether raw metabolomics tandem mass spectra can serve as pathogen biomarkers (8).

Sugarcane is another crop of interest in Brazil, as the raw product can be used to produce sugar, bioethanol, and silage and as biomass, among other purposes. Given the use of mechanization in Brazil during the past 5 to 7 years, techniques employing controlled fire are no longer used during preharvest. Despite the positive effects for the environment and workers, new problems have emerged. There are growing problems with browning of sugarcane juice which could be related to an increase of humidity content and microbial load in sugarcane prior to fermentation. We are using metabolomics to study mechanisms involved in the browning of the sugarcane tissues under conditions of biotic and/or abiotic stresses and to determine whether we are able to pinpoint the microbes or pests that are involved in such feature. We are keen to propose corrective measures for the continuous improvement of the quality of the white sugar produced in Brazil, noting that Brazilian sugar production accounts for about 30% of the sugar consumed globally. Another problem caused by the absence of fire during the preharvest is the control of *Mahanarva fimbriolata*. The nymph secretes a white froth, usually in the bottom part of the plant, which acts as a physical barrier against chemical control. We are evaluating the metabolome of the froth, as this can indicate its function and the chemical pathway involved in the froth production and thus might provide a solution to the problem.

We are a young group whose interest lies in applying metabolomics to tackle, mainly, agricultural issues, as Brazil has specific issues which have never been fully assessed before. Apart from what was briefly presented, we also intend to prospect natural products from Brazilian distinct biomes, as Brazilian natural resources are yet to be fully explored. Thus, we are excited about the opportunity to study and comprehend system-wide metabolism and metabolic pathways, which can have direct and indirect impacts on regional and global problems. Finally, we strongly believe that working with a multidisciplinary team, with regional and global collaborators, will be key to facing global challenges.
